# Clinical Impact of Molecular Subtyping of Pancreatic Cancer

**DOI:** 10.3389/fcell.2021.743908

**Published:** 2021-11-05

**Authors:** Xu Zhou, Kai Hu, Peter Bailey, Christoph Springfeld, Susanne Roth, Roma Kurilov, Benedikt Brors, Thomas Gress, Malte Buchholz, Jingyu An, Kongyuan Wei, Teresa Peccerella, Markus W. Büchler, Thilo Hackert, John P. Neoptolemos

**Affiliations:** ^1^Department of General, Visceral and Transplantation Surgery, Heidelberg University Hospital, Heidelberg, Germany; ^2^Section of Surgical Research, Heidelberg University Hospital, Heidelberg, Germany; ^3^Institute of Cancer Sciences, University of Glasgow, Glasgow, United Kingdom; ^4^Department of Medical Oncology, National Center for Tumor Diseases, Heidelberg University Hospital, Heidelberg, Germany; ^5^Division of Applied Bioinformatics, German Cancer Research Center, Heidelberg, Germany; ^6^Department of Gastroenterology and Endocrinology, Philipps University of Marburg, Marburg, Germany

**Keywords:** molecular subtypes, transcriptomes, structural variants, precision medicine, next generation sequencing, clinical trials, ESPAC

## Abstract

Pancreatic ductal adenocarcinoma is a highly lethal malignancy, which has now become the seventh most common cause of cancer death in the world, with the highest mortality rates in Europe and North America. In the past 30 years, there has been some progress in 5-year survival (rates increasing from 2.5 to 10%), but this is still extremely poor compared to all other common cancer types. Targeted therapies for advanced pancreatic cancer based on actionable mutations have been disappointing, with only 3–5% showing even a short clinical benefit. There is, however, a molecular diversity beyond mutations in genes responsible for producing classical canonical signaling pathways. Pancreatic cancer is almost unique in promoting an excess production of other components of the stroma, resulting in a complex tumor microenvironment that contributes to tumor development, progression, and response to treatment. Various transcriptional subtypes have also been described. Most notably, there is a strong alignment between the Classical/Pancreatic progenitor and Quasi-mesenchymal/Basal-like/Squamous subtype signatures of Moffit, Collinson, Bailey, Puleo, and Chan-Seng-Yue, which have potential clinical impact. Sequencing of epithelial cell populations enriched by laser capture microscopy combined with single-cell RNA sequencing has revealed the potential genomic evolution of pancreatic cancer as being a consequence of a gene expression continuum from mixed Basal-like and Classical cell populations within the same tumor, linked to allelic imbalances in mutant KRAS, with metastatic tumors being more copy number-unstable compared to primary tumors. The Basal-like subtype appears more chemoresistant with reduced survival compared to the Classical subtype. Chemotherapy and/or chemoradiation will also enrich the Basal-like subtype. Squamous/Basal-like programs facilitate immune infiltration compared with the Classical-like programs. The immune infiltrates associated with Basal and Classical type cells are distinct, potentially opening the door to differential strategies. Single-cell and spatial transcriptomics will now allow single cell profiling of tumor and resident immune cell populations that may further advance subtyping. Multiple clinical trials have been launched based on transcriptomic response signatures and molecular subtyping including COMPASS, Precision Promise, ESPAC6/7, PREDICT-PACA, and PASS1. We review several approaches to explore the clinical relevance of molecular profiling to provide optimal bench-to-beside translation with clinical impact.

## Introduction

Pancreatic ductal adenocarcinoma (PDAC), a distinct form of pancreatic cancer, remains a major oncological challenge (Kleeff et al., 2016). Globally the 5-year pancreatic cancer prevalence in 2020 was 4.87 per 10^5^ per year (International Agency for Research on Cancer, 2021). The number of cases of pancreatic cancer worldwide in 2020 was 495,773 (world rank for all cancers = 13), with 466,003 deaths (world rank for all cancers = 7); incidence rates per 10^5^ per year were 5.7 for men and 4.1 for women, and mortality rates of 4.9 and 4.5, respectively (International Agency for Research on Cancer, 2021). In Europe, there were 140,116 new cases with 132,134 deaths (International Agency for Research on Cancer, 2021). In North America, there were 62,643 new cases and 53,277 deaths, the fourth highest cancer mortality in both men and women (International Agency for Research on Cancer, 2021; Siegel et al., 2021). For Western Europe, the incidence rates per 10^5^ per year were 9.9 for men and 7.4 for women, with mortality rates of 8.6 and 7.8, respectively (world rank first for pancreatic cancer) (International Agency for Research on Cancer, 2021). In 2017, in Germany, there were 18,687 new cases (with a rising incidence rate) and 18,005 deaths, but with a slight improvement in the 5-year survival rate from 8% in 2007–2008 to 9% in 2015–2016 (Zentrum für Krebsregisterdaten, 2021). In the United States, the 5-year survival rate for all stages has further improved to 10% (Siegel et al., 2021).

## Standard Therapies for Pancreatic Cancer

Most patients present with metastatic disease, with only 10–20% being diagnosed with localized pancreatic cancer that can be surgically removed, while the remaining 20–30% have non-metastatic locally advanced disease that cannot be removed by standard surgical techniques (Kleeff et al., 2016). Systemic chemotherapy is the only conventional approach for improving survival in patients with advanced disease with the best achievable median survival rates being 8–12 months for metastatic disease and 12–15 months for locally advanced pancreatic cancer (Burris et al., 1997; Cunningham et al., 2009; Conroy et al., 2011; Von Hoff et al., 2013; Gill et al., 2016; Wang-Gillam et al., 2016; Springfeld et al., 2019). Although chemoradiotherapy is often used for locally advanced disease especially in the United States, there is increased toxicity without improvement in overall survival (Sultana et al., 2007; Chauffert et al., 2008; Hammel et al., 2016). In patients with locally resectable tumors but without metastatic disease, advances in surgical techniques and the use of adjuvant systematic chemotherapy have increased 5-year survival rates from 8% with resection alone to 30–50% in conjunction with adjuvant chemotherapy most notably using gemcitabine and capecitabine or modified folinic acid, 5-fluorouracil (5-FU), irinotecan, and oxaliplatin (mFOLFIRINOX) combinations (Neoptolemos et al., 2004; Oettle et al., 2007; Neoptolemos et al., 2010; Kleeff et al., 2016; Uesaka et al., 2016; Neoptolemos et al., 2017; Conroy et al., 2018; Strobel et al., 2019). Patients with borderline resectable disease may benefit from neoadjuvant chemotherapy regimens comprising gemcitabine with capecitabine as well as mFOLFIRINOX, while regimens with chemoradiotherapy are inferior to chemotherapy alone (Ghaneh et al., 2020; Katz et al., 2021). Neoadjuvant therapy may also increase resectabilty with improved survival in patients with otherwise unresectable local disease due to major vessel encasement using comprising mFOLFIRINOX or gemcitabine-based regimens with either capecitabine or nab-paclitaxel (Hackert et al., 2016; Diener et al., 2021; Kunzmann et al., 2021). An argument has been made to extend the use of neoadjuvant chemotherapy to patients with resectable disease, but this appears to be inferior for overall survival compared to upfront surgery and adjuvant treatment (Sohal et al., 2021).

The evolution of molecular targeted therapies, aimed at advancing tumor control and cell killing of pancreatic cancer, has so far met with only very limited progress (Davis et al., 2019; Golan et al., 2019; Mosele et al., 2020; O’Kane et al., 2020; Pishvaian et al., 2020; Cobain et al., 2021). While systemic chemotherapy is the mainstay of treatment when added to surgery, its impact is limited by a wide variation in responsiveness that is related to intrinsic and acquired mechanisms of sensitivity and resistance by both the cancer cells themselves and the stromal environment (Greenhalf et al., 2014; Martinez-Balibrea et al., 2015; Noll et al., 2016; Amrutkar and Gladhaug, 2017; Geller et al., 2017; Martinelli et al., 2017; Schlitter et al., 2017; Neoptolemos et al., 2018; Tiriac et al., 2018; Dominguez et al., 2020; Kalimuthu et al., 2020; Sahai et al., 2020). Going one step further, the integration of molecular subtypes derived from global genomic and transcriptomic analyses into clinical trials is enabling translational insights into how we might better refine existing and evolving therapy modalities to improve pancreatic cancer treatment. Pancreatic cancer is almost unique in promoting an excess production of other components of the admixture of general tissue (stroma), resulting in a complex tumor microenvironment that contributes to tumor development, progression, and response to treatment (Kleeff et al., 2016).

## Single Gene Alterations in Pancreatic Cancer

Mutations in genes responsible for producing classical canonical signaling pathways including driver oncogenes and dysfunction of tumor suppressor genes include KRAS, TP53, CDKN2A, and SMAD4 in most cases, and ARID1A, KDM6A, MLL3, TGFBR2, RBM10, BCORL1, and ROBO2 in 5–10% of tumors (Jones et al., 2008; Waddell et al., 2015; Bailey et al., 2016; Chan-Seng-Yue et al., 2020; Escobar-Hoyos et al., 2020). Genetic alterations occur in each of a core set of 12 cellular signaling pathways in 67–100% of the tumors, with representative genes listed below (Jones et al., 2008).

• Apoptosis: CASP10, VCP, CAD, HIP1
• DNA damage control: ERCC4, ERCC6, EP300, RANBP2, TP53, BRCA1/2, PALB2, ATM, ATR, MLH1, MSH2, MSH6, RPA1, STK11, FANCA, FANCC
• Regulation of G1/S phase transition: CDKN2A, FBXW7, CHD1, APC2
• Hedgehog signaling: TBX5, SOX3, LRP2, GLI1, GLI3, BMPR2, CREBBP
• Homophilic cell adhesion: CDH1, FAT, PCDH15, PCDHB16, PCDHGA1
• Integrin signaling: ITGA4, LAMA1, LAMA4, LAMA5, FN1, ILK
• c-Jun N-terminal kinase signaling: MAP4K3, TNF, ATF2, NFATC3
• KRAS signaling: KRAS, MAP2K4, RASGRP3
• Regulation of invasion: ADAM11, DPP6, MEP1A, PCSK6, APG4A
• Small GTPase–dependent signaling: AGHGEF7, ARHGEF9, CDC42BPA
• TGF-β signaling: TGFBR2, BMPR2, SMAD4, SMAD3
• Wnt/Notch signaling: MYC, PPP2R3A, WNT9A, MAP2, TSC2, GATA6.

Single genetic alterations occur in <5% of tumors, notably BRCA1/2 mutations, BRAF gene fusions/mutations, ERBB2 amplifications/mutations, RNF43, TGFBR2, MAP2K4, MLL3, PIK3CA, RBM10, SMARCA4, PBRM1, SLIT2, KDM6A, GATA6, BRAF, ATM, and mismatch repair (MMR) gene mutations (Waddell et al., 2015). MMR genes (MLH1, MSH2, MSH6, and PMS2) normally recognize mistakes in insertion, deletion, or mismatched incorporation of nucleotides arising from errors by DNA polymerases and then replacing them with the correct nucleotides. As well as gene mutations, loss of MMR protein function may arise through promoter methylation especially in the case of MLH1. The consequence is an accumulation of errors in DNA microsatellites (short repetitive sequences in DNA) causing high microsatellite instability (MSI-H) (Table 1; Waddell et al., 2015; Mosele et al., 2020).

**TABLE 1:** List of actionable single gene alterations to in advanced pancreatic ductal adenocarcinoma in accordance with ESMO Scale for Clinical Actionability of Molecular Targets (ESCAT) levels I–III (Mateo et al., 2018; Mosele et al., 2020).

**Gene**	**Alteration**	**Prevalence**	**ESCAT Level**
BRCA1/2	Germline mutations	1–4%	I
BRCA1/2	Somatic mutations	3%	III
MSH1, PMS2, MLH1, and MSH6	MSI-H	1–3%	I
NTRK	Fusions	<1%	I
KRAS^*G12C*^	Mutation	1–2%	II
PIK3CA	Hotspot mutations	3%	III
BRAF^V600E^	Mutations	3%	III
MDM2	Amplifications	2%	III
ERBB2	Amplifications/mutations	1–2%	III
NRG1	Fusions	1%	III
ALK	Fusions	<1%	III
RET	Fusions	<1%	III
ROS1	Fusions	<1%	III

Clinical applicability of genetic biomarkers has been classified by the European Society for Medical Oncology (ESMO) Translational Research and Precision Medicine Working Group into the ESMO Scale of Clinical Actionability for molecular Targets (ESCAT) (Mateo et al., 2018). There are four main levels defined as follows: I = the match of an alteration and a drug has been validated in clinical trials, and should drive treatment decision in daily practice; II = a drug that matches the alteration has been associated with responses in phase I/II or in retrospective analyses of randomized trials; III = alterations that are validated in another cancer, but not in the disease-to-treat; IV = hypothetically targetable alterations based on preclinical data (Mateo et al., 2018; Mosele et al., 2020). So far, the clinical utility of targeting drugs to specific molecular alterations is rather limited (Hong et al., 2020; Mosele et al., 2020):

**mFOLFIRINOX** –preferred for known germline BRCA1/2 or PALB2 mutations.
**Olaparib** – a PARP inhibitor as maintenance therapy in patients who have a germline BRCA1 or BRCA2 mutation and with metastatic pancreatic cancer that had not progressed during first-line platinum-based chemotherapy, resulting in improved progression-free survival.
**Entrectinib** – an inhibitor of tropomyosin receptor kinases (TRKs) of tumors with NTRK or ROS-1 gene fusions.
**Laroctrenib** – an inhibitor of tropomyosin receptor kinases (TRKs) of tumors with NTRK gene fusions.
**Afatinib** – an EGFR tyrosine kinase inhibitor in KRAS wild-type tumors with NRG1 gene fusions.
**Sotorasib** – a small molecule that targets the KRAS p.G12C mutation that is present in 1–2% of PDAC patients (Hong et al., 2020).

Also, erlotinib, a multiple tyrosine kinase inhibitor (including EGFR) used with gemcitabine, produces an improved survival in metastatic pancreatic cancer, but this benefit is only marginal with increased toxicity.

## Actionable Genomic Subtypes

Structural variations amongst the 25,000 defined human genomes include deletions, amplifications, duplications, and translocations (International Cancer Genome Consortium et al., 2010; Waddell et al., 2015). The Waddell signature based on whole-genome sequencing and copy number variation identified four subtypes based on patterns of chromosomal structural variation with potential clinical utility (Table 2; Waddell et al., 2015).

**TABLE 2:** Main molecular subtypes of pancreatic cancer.

**Study name**	**Year**	**Sample source and number**	**Methodology**	**Tumor cellularity**	**Source of the DNA/RNA**	**Subtyping method**	**Molecular subtypes**	**Promising molecular biomarkers**	**Clinical relevance**
Waddell et al., 2015	2015	Primary resected tumors (*n* = 100)	WGS		Cryo bulk tissue	Structural rearrangements	Stable, locally rearranged, scattered, unstable	BRCA mutation (frequent in unstable tumors)	Unstable genome and/or BRCA mutation tumors responded well to platinum-based therapy.
Collisson et al., 2011	2011	Primary resected PDAC (*n* = 27); PDXs (*n* = 19); Mouse cell lines (*n* = 15)	Gene expression microarray	High	Cryo samples underwent microdissection	Tumor transcriptional profiles	Classical, QM-PDA, exocrine-like	GATA6 (high in classical and low in QM-PDA)	QM-PDA subtype was correlated with poor outcome and was sensitive to gemcitabine; classical subtype was correlated with good outcome and was sensitive to erlotinib.
Moffitt et al., 2015	2015	Primary (*n* = 145) and metastatic (*n* = 61) PDAC; Cell lines (*n* = 17); Pancreas (*n* = 46) and distant adjacent normal samples (*n* = 88); PDXs (*n* = 37); CAF (*n* = 6)	Whole-genome DNA microarrays, RNA-seq, virtual microdissection		Cryo bulk tissue, cell lines, PDXs, CAFs	Tumor and stroma transcriptional profiles	Tumor: classical, basal-like Stroma: normal, activated	SMAD4 and GATA6 (high in classical and low in basal-like)	Classical tumors with normal stroma had best outcome and basal-like tumors with activated stroma had worst outcome; basal-like tumors responded to adjuvant therapy better than classical tumors.
Bailey et al., 2016	2016	Primary resected tumors (*n* = 456); Patient-derived cell lines (*n* = 41); Mouse cell lines	WGS, deep-exome sequencing, RNA-seq	>40% for WGS, 12–40% for deep-exome sequencing	Cryo bulk tissue	Tumor transcriptional profiles	Squamous, pancreatic progenitor, immunogenic, ADEX	TP53 and KDM6A mutations (frequent in squamous tumors); FOXA2/3, PDX1 and MNX1 (high in progenitor tumors)	Squamous tumors had worst outcome.
Raphael and Cancer Genome Atlas Research Network, 2017	2017	Primary resected PDAC (*n* = 150)	WES, custom targeted gene panel sequencing, RNA-seq		Cryo bulk tissue	Tumor transcriptional profiles	Validation of former classifications (Moffitt’s classification was independent of tumor purity, while Collisson’s and Bailey’s classifications were correlated with tumor purity)	Low EMT and apoptosis pathway activity, high TSC-mTOR and RTK activity in better survival groups	
Wartenberg et al., 2018	2018	Primary resected PDAC (*n* = 110)	ngTMA		FFPE samples	Tumor transcriptional profiles	Immune escape, immune rich and immune exhausted		Immune escape tumors had poor outcome, and immune rich tumors had better outcome
Puleo et al., 2018	2018	Primary resected PDAC (*n* = 381)	RNA microarray, immunohistochemistry, DNA panel sequencing		FFPE samples	Tumor transcriptional profiles	Pure classical, immune classical, desmoplastic, stroma activated, pure basal-like		Pure basal-like tumors had the worst outcome.
Maurer et al., 2019	2019	Primary resected PDAC (*n* = 60)	RNA-seq	High	Cryo samples underwent LCM	Tumor and stroma transcriptional profiles	Tumor: classical, basal-like Stroma: immune-rich, ECM-rich		Basal-like/ECM-rich tumors had worse outcome than classical/immune-rich tumors
Dijk et al., 2020	2020	Primary resected PDAC (*n* = 90)	RNA-seq		Cryo bulk tissue, PDXs	Tumor transcriptional profiles	Secretory, epithelial, compound pancreatic and mesenchymal	KRAS (highest transcript level in mesenchymal subtype)	Mesenchymal and secretory tumors had worse outcome than epithelial and compound pancreatic tumors.
Chan-Seng-Yue et al., 2020	2020	Primary resected PDAC (*n* = 206) and advanced PDAC (*n* = 111)	WGS, RNA-seq, single cell analysis	High	Cryo samples underwent LCM, single cell	Tumor transcriptional profiles	Basal-like-A, basal-like-B, hybrid, classical-A, classical-B	SMAD4 (high in basal-like-A tumors and low in classical-A tumors), CDKN2A and TP53 (completely loss in basal-like-A/B tumors), GATA6 (high in Classical-A/B tumors)	Classical-A/B tumors were more frequent in early stage, while basal-like tumors in late stage; in resectable disease, basal-like-B and hybrid tumors identified two prognostic subgroups considered to be uniformly aggressive before; in advanced disease, basal-like-A was highly chemo-resistant and trended toward worse survival.

**Stable (20%)**, with <50 structural variations per genome, with widespread aneuploidy.
**Locally rearranged (30%)**, with >200 structural variants clustered on 1–2 chromosomes. Of these, about a 35% had focal amplifications in KRAS, SOX9 and GATA6, as well as ERBB2, MET, CDK6, PIK3CA, and PIK3R3 but were only present in 1–2% of patients. The remaining local rearrangements involved complex genomic events such as breakage–fusion–bridge or chromothripsis (thousands of clustered chromosomal rearrangements occurring in a single event in localized and confined genomic regions in one or two chromosomes).
**Scattered (36%)**, with non-random chromosomal damage in 50–200 structural variants per genome.
**Unstable (14%)**, with >200 structural variants distributed across the genome indicating defects in DNA maintenance (BRCA1/2, and PALB2 gene defects) and a mutational DNA damage repair (DDR) deficiency, with potential sensitivity to DNA-damaging agents. The unstable structural variation subtype is responsive to platinum therapy and BRCA1/2 germline carriers also sensitive to both platinum and PARP inhibitors.

It is estimated that 24% of all pancreatic cancers may be sensitive to platinum therapy based on an unstable genomic structural variation subtype, and/or somatic and germline mutations in BRCA genes, and/or a BRCA-type mutational signature (Waddell et al., 2015). MSI-H occurs in 1–3% of pancreatic cancers, which is commonly associated with mutations in the MSH2 and MLH1 MMR genes, and can be detected by immunohistochemistry (MSH1, PMS2, MLH1, and MSH6 expression) or sequencing (single gene mutations and MMR mutational signature) (Waddell et al., 2015; Connor et al., 2017).

MSI-H tumors express a large number of neoantigens, potentially rendering them more susceptible to immunotherapy in comparison to those tumors with relatively few mutations. DNA replication stress producing single-stranded DNA will induce DDR of which the DNA damage checkpoint kinase ATR [Ataxia-Telangiectasia Mutated (ATM) and Rad3-related protein kinase] is a critical component. Genes encoding subunits of SWI/SNF (BAF) chromatin remodeling complexes, each composed of approximately 15 protein subunits, include ARID1A, ARID1B, ARID2, PBRM1, SMARCA4, and SMARCB1. ARID1A deficiency will impair cells to recruit topoisomerase 2A to chromatin causing cell cycle defects. The consequence is increased reliance on ATR checkpoint activity and thereby increased sensitivity to ATR inhibitor therapy (Williamson et al., 2016).

## Transcriptomic Subtypes

Various transcriptional pancreatic cancer subtypes have also been described, most notably the Moffit, Collinson, Bailey, Puleo, and Chan-Seng-Yue signatures amongst others, which have potential clinical impact (Table 2; Collisson et al., 2011; Moffitt et al., 2015; Bailey et al., 2016; Cancer Genome Atlas Research Network, 2017; Puleo et al., 2018; Wartenberg et al., 2018; Maurer et al., 2019; Chan-Seng-Yue et al., 2020; Dijk et al., 2020). Each study has used a different approach to deal with the low cellularity and stromal contribution, leading to some debate regards the value of those subtypes. Two dominant transcriptional subtypes have emerged: a Classical subtype that tends to be more responsive to chemotherapy and a very aggressive poorly differentiated Squamous/Basal-like subtype.

Collisson et al. (2011) used micro-dissected tumor samples from resected primary PDAC from two different clinical series to define three specific gene expression subtypes.

**Exocrine-like:** characterized by relatively high expression of tumor cell derived digestive enzyme genes.
**Classical:** demonstrating high expression of adhesion-associated and epithelial genes, and epithelial cell terminal differentiation genes, notably GATA6; KRAS mRNA levels elevated relative to the other subtypes; Classical subtype cell lines are more sensitive to erlotinib.
**Quasi-mesenchymal:** has high expression of mesenchyme associated genes; a relatively high proportion of high-grade tumors and poor patient outcomes; low GATA6 expression; QM-PDA subtype cell lines are relatively more sensitive to gemcitabine than those with the Classical subtype.

Moffitt et al. (2015) used a diverse collection of pancreatic gene expression microarray data, including normal pancreata samples as well as primary and metastatic cancer samples, to identify two tumor-specific subtypes as well as additional stromal Normal and Activated subtypes which were independently prognostic. To develop their two tumor-specific subtypes, Moffitt et al. excluded transcripts thought to be specifically enriched in either the normal pancreas or the tumor microenvironment. The two tumor-specific subtypes were referred to as Classical and Basal-Like (Moffitt et al., 2015).

**Classical:** characterized by overlapping signature with the genes described in the Collisson classification including GATA6, and overall a better prognosis.
**Basal-like:** associated with a worse prognosis than the Classical subtype but may have a better response to adjuvant therapy.

Bailey et al. (2016) described four subtypes using samples with >40% cellularity from resectable primary pancreatic cancer, based on differential transcription factor expression and downstream targets responsible for lineage specification and differentiation during development and regeneration.

**Squamous:** is characterized by enrichment for TP53 and KDM6A mutations; upregulation of the TP63ΔN transcriptional network; hypermethylation of pancreatic endodermal cell-fate determining genes and is associated with a poor clinical prognosis.
**Pancreatic progenitor:** is defined by preferential expression of genes involved in early pancreatic development notably FOXA2/3, PDX1, and MNX1 and also by gene programs involved in metabolism.
**Immunogenic:** is classed by the enrichment of genes associated with specific immune cell populations, including T-cells and B-cells.
**Aberrantly differentiated endocrine exocrine (ADEX):** is featured by upregulation of genes that regulate networks involved in KRAS activation, and exocrine (NR5A2 and RBPJL) and endocrine differentiation (NEUROD1 and NKX2-2).

The Squamous subtype overlaps with the Quasi-mesenchymal subtype of Collisson but has notable pan-squamous features, including a significant association with adenosquamous PDAC histology (Bailey et al., 2016). There is a marked epigenetic shift, with changes in DNA methylation down-regulating key transcription factors controlling pancreatic cell fate determination (PDX1, MNX1, GATA6, HNF1B), and the activation of subtype-driver multigene programs regulated by ΔNTP63 and c-MYC, leading to a loss of endodermal identity (Bailey et al., 2016). In addition, the Squamous subtype was also found to be enriched for mutations in KDM6A, MLL2, and MLL3 chromatin modifying enzymes that belong to the COMPASS complex (COMplex of Proteins Associated with Set1-like) (Bailey et al., 2016).

The Pancreatic progenitor subtype has four key characteristics.

***(i) Transcriptional networks*** containing transcription factors PDX1, MNX1, HNF4G, HNF4A, HNF1B, HNF1A, FOXA2, FOXA3, and HES1, which are pivotal for pancreatic endoderm cell-fate determination toward a pancreatic lineage and are linked to maturity onset diabetes of the young
***(ii) Gene programs regulating metabolism*** notably fatty acid oxidation, steroid hormone biosynthesis, and drug metabolism
***(iii) O-linked glycosylation of mucins***, notably apomucins MUC5AC and MUC1, but not MUC2 or MUC6, that define the IPMN pancreatobiliary subtype with PDAC-associated IPMN clustering
***(iv) TGFBR2 inactivating mutations.***

The ADEX subtype was defined by both exocrine and endocrine lineage features in later stages of pancreatic development and differentiation (rather than one or the other as is in normal pancreas development), and could be considered a subclass of Pancreatic progenitor tumors. There are two main transcriptional networks.

***(i) Acinar cell differentiation and pancreatitis/regeneration***, transcription factors NR5A2, MIST1 (BHLHA15A) and RBPJL and their downstream targets.
***(ii) Endocrine differentiation and maturity onset diabetes of the young***, including INS, NEUROD1, NKX2-2, and MAFA.

Puleo et al. (2018) proposed two classifications, one specifically for the transformed neoplastic tumor cells and the other for the complete tumor entity, including the stroma: pure basal-like, stroma activated, desmoplastic, pure classical, and immune classical:

**Pure basal-like** tumors are composed of poorly differentiated tumors with predominant Gly12Asp and Gly12Val KRAS mutations; they have a low stromal signal.
**Stroma Activated** tumors are moderately differentiated, specifically enriched in the activated stroma component defined by high a-SMA, SPARC, and FAP.
**Desmoplastic** tumors are also moderately differentiated with a predominant basal association, characterized by a low tumoral component and a large stromal transcriptomic signal, including immune and inflammatory stroma components and, particularly, a high expression of structural and vascularized stroma components.
**Pure-classical and Immune classical** tumors are histologically well differentiated with fewer CDKN2A and TP53 mutations than basal-like tumors, and are also enriched with the Gly12Arg KRAS mutation, and associated with hENT1 expression; predicted to be Moffitt–Classical, and Bailey–Progenitor subtypes.

Maurer et al. used laser capture microdissection (LCM) epithelial cell enriched samples for mRNA sequencing to profile the expression of 60 matched pairs of human PDAC malignant epithelial and stroma samples (Maurer et al., 2019). They developed a computational model that could infer tissue composition and generate virtual compartment-specific expression profiles from bulk gene expression cohorts (Maurer et al., 2019). This study was able to provide a clearer understanding on the previous molecular gene signatures built from bulk tumor tissue samples with the following conclusions.

(1) Genes used to define the Collisson–Classical, Moffitt–Classical, Moffitt–Basal-like, and Bailey–Progenitor subtypes predominantly provide information about the malignant compartment regardless of the amount of stromal cell infiltration.
(2) Genes used to define the Moffitt–Activated, Moffitt–Normal, and Bailey–Immunogenic subtypes report on stromal expression that is largely independent of the malignant compartment (but see below).
(3) Gene sets in the Collisson–Quasi-Mesenchymal and Bailey–Squamous subtypes represent a mixture of epithelial and stromal identity, indicative of a more poorly differentiated state.
(4) Most genes that define the Collisson–Exocrine and Bailey–ADEX subtypes are largely derived from bulk tumor tissue samples are arguably mostly absent from LCM samples (but see below).

Chan-Seng-Yue et al. (2020) used LCM-purified pancreatic cancers for whole-genome sequencing in tumors from 314 patients, and whole-transcriptome sequencing of tumors from 248 patients, accompanied by single-cell RNA sequencing on 13 resectable and two metastatic tumors. For this classification, tumors with homologous recombination defects and MMR deficiency were excluded due to their unique mutational signatures (Chan-Seng-Yue et al., 2020).

**Basal-like A:** these tumors were associated with the epithelial mesenchymal transition (EMT) program; TP53 gene and TGF-β signaling enriched; 5% of stage I/II (resectable); and 24% of stage IV (metastatic) tumors.
**Basal-like B:** these tumors were associated with the EMT program; TP53 and TGF-β signaling enriched; 9% of stage I/II (resectable), 7% of stage III (locally advanced), and 12% of stage IV (metastatic) tumors.
**Hybrid:** this subtype was found in 24% of stage I/II (resectable), 43% of stage III (locally advanced), and 18% of stage IV (metastatic) tumors.
**Classical A:** this subtype was found in 44% of stage I/II (resectable), 43% of stage III (locally advanced), and 36% of stage IV (metastatic) tumors.
**Classical B:** this subtype was found in 8% of stage I/II (resectable), 7% of stage III (locally advanced), and 10% of stage IV (metastatic) tumors.

This classification split each of the previously defined Basal-like and Classical subtypes into two disease subtypes, while the Hybrid subtype was inconsistently classified by previous systems arising from multiple expression profiles (Chan-Seng-Yue et al., 2020). Single-cell RNA sequencing revealed that both Basal-like and Classical clusters were present in the same tumor found in 13 out of 15 patients (Chan-Seng-Yue et al., 2020). The EMT program was positively correlated with Basal-like signatures and negatively correlated with Classical signatures (Chan-Seng-Yue et al., 2020). Moreover, they found that a major imbalance of allelic states of KRAS (KRAS^*Ma*^) favoring the mutant allele over the wild-type allele occurred in only 4% of primary tumors compared to 29% in metastatic disease (Chan-Seng-Yue et al., 2020). Basal-like A/B tumors were enriched for the major imbalance KRAS^*Ma*^ allelic states (44%) compared to metastatic Classical A/B tumors (14%), and KRAS^*Ma*^ tumors were also more chemoresistant (Chan-Seng-Yue et al., 2020).

They proposed a possible model for the genomic evolution of pancreatic cancer as being a consequence of a gene expression continuum from (a) both Basal-like and Classical cell populations, and (b) linked to allelic imbalances in mutant (mt) KRAS, with metastatic tumors being more copy number-unstable compared to primary tumors (Chan-Seng-Yue et al., 2020). In primary tumors, the Basal-like phenotype is linked to minor mtKRAS allelic imbalances, whist in metastatic tumors, it is linked to major mtKRAS allelic imbalances (Chan-Seng-Yue et al., 2020).

### Potential Influence of Tumor Cellularity on Transcriptomic Subtypes

Raphael and Cancer Genome Atlas Research Network (2017) performed genomic, transcriptomic, and proteomic profiling of 150 PDAC specimens, including samples with low neoplastic cellularity, provided by the Cancer Genome Atlas Research Network. They applied clustering techniques to reproduce the four-group classification of Bailey et al. (2016; Squamous, Immunogenic, Pancreatic Progenitor, and ADEX), the three-group classification (Classical, Quasi-mesenchymal, and Exocrine-like) of Collisson et al. (2011) and the two-group classification (Basal-like or Classical) of Moffitt et al. (2015). They found that while the Basal-like and Classical subtypes were independent of cancer cell purity, the Collisson Exocrine-like and Quasi-Mesenchymal subtypes, and the Bailey ADEX and Immunogenic subtypes were all associated with lower tumor purity (Raphael and Cancer Genome Atlas Research Network, 2017). Raphael and Cancer Genome Atlas Research Network (2017) also found that, among low purity tumors, a higher estimated leukocyte fraction was associated with the Immunogenic subtype and that the ADEX subtype was a subset of the Collisson Exocrine-like subtype.

Puleo et al. (2018) using formalin-fixed and paraffin-embedded tissues also concluded that the ADEX tumor subtype largely resulted from contamination with pancreatic acinar cells. As few as 39 of the most highly expressed genes of normal acinar cells from healthy pancreas single-cell transcriptomes can alone constitute 50% of the total number of expressed transcripts such that even a low level of normal pancreas contamination can materially affect any otherwise presumed subtype (Puleo et al., 2018).

Maurer et al. (2019) also suggested that the Collisson–Exocrine and Bailey–ADEX subtypes might be a function of the degree of tumor cellularity rather than being a distinct subtype as most of the subtype defining genes are largely derived from bulk tumor tissue samples and are mostly absent from LCM epithelial cell enriched samples.

**FIGURE 1:**
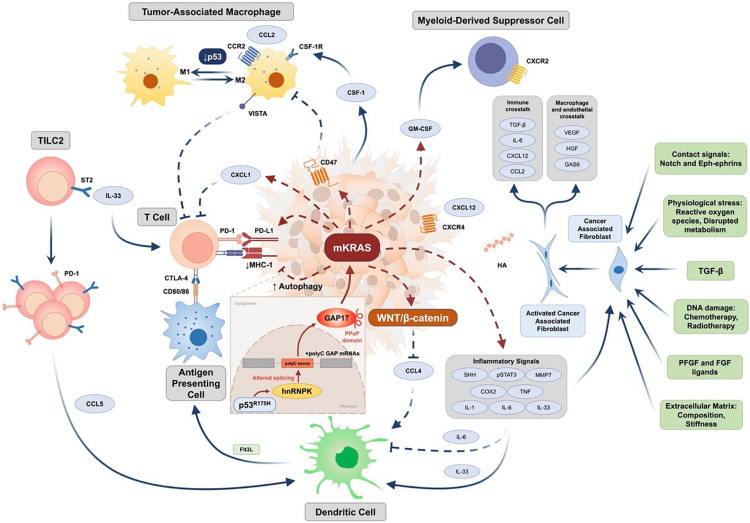
Epithelial and stromal cell interactions in pancreatic cancer (Jones et al., 2008; Waddell et al., 2015; Bailey et al., 2016; Bear et al., 2020; Dominguez et al., 2020; Escobar-Hoyos et al., 2020; Sahai et al., 2020). PDAC immune resistance is driven by complex genetic background. Expression of tumor-intrinsic GM-CSF and CXCL1 is increased by oncogenic KRAS to mediate T cell exclusion and MDSC infiltration. Downstream signaling initiated by mutant KRAS (mKRAS) mediates innate and adaptive immune escape through enhancing autophagy to downregulate MHC-1 expression and upregulate the expression of PD-L1 and CD47. In addition to increased IL-6-mediated systemic dysregulation of conventional type 1 dendritic cell (DC), activation of WNT/β-catenin mediated by mKRAS signaling further downregulates CCL4 expression to inhibit DC recruitment. Tumor group 2 innate lymphoid cells (TILC2s) infiltrate the tumor microenvironment and are activated by IL-33 through binding to the ST2 receptor, further leading to an enhancement of anti-tumor immunity by expressing the inhibitory checkpoint receptor PD-1 and recruiting DCs potentially through CCL5 production. Furthermore, mKRAS signaling enhances chronic inflammation signaling such as Sonic Hedgehog, COX2, and pSTAT3 signaling, and promotes multiple inflammation-associated factors such as IL-1, IL-6, tumor necrosis factor (TNF), and matrix metalloproteinase 7 (MMP) to activate cancer-associated fibroblasts (CAF). Additional factors leading to activation of CAFs include TGF-β, extracellular matrix (ECM) stiffness and composition, RTK ligands such as PDGF and FGF, DNA damage caused by chemotherapy and radiotherapy, physiological stress, and contact signals such as Notch and Eph-ephrins. Activated CAFs further regulate macrophage and endothelial functions by factors such as VEGF, HGF, and GAS6 and participate in immune crosstalk through TGF-β activation, IL-6, CXCL12, and CCL2 production. Deficiency of p53 mediates transition of TAM toward an immunosuppressive M2 phenotype. Mutant p53 (such as R175H) increases expression of the splicing regulator hnRNPK to promote inclusion of cytosine-rich exons (+polyC exons) within GTPase-activating proteins (GAPs), particularly GAP17, leading to enhanced KRAS activity. CCL2/4/5, CC-chemokine ligand 2/4/5; CCR, CC-chemokine receptor; COX2, cyclooxygenase 2; CSF-1, colony-stimulating factor 1; CSF-1R, colony stimulating factor 1 receptor; CTLA-4, cytotoxic T-lymphocyte-associated protein 4; CXCL1/12, CXC-chemokine ligand 1/12; CXCR4, CXC-chemokine receptor type 4; DC, conventional type 1 dendritic cell; FGF, fibroblast growth factor; Flt3L, Fms related receptor tyrosine kinase 3 ligand; GAS6, growth arrest-specific protein 6; GM-CSF, granulocyte-macrophage colony-stimulating factor; hnRNPK, heterogeneous nuclear ribonucleoprotein K; HA, hyaluronic acid; HGF, hepatocyte growth factor; IL-1/-6/-33, interleukin-1/-6/-33; MHC-1, major histocompatibility complex 1; PDGF, platelet-derived growth factor; PD-1, programmed cell death protein 1; PD-L1, programmed death-ligand 1; SHH, sonic hedgehog; ST2, suppression of tumorigenicity 2; STAT3, signal transducer and activator of transcription 3; TAM, tumor-associated macrophage; TGF-β, transforming growth factor-β; VEGF, vascular endothelial growth factor; VISTA, V-domain Ig suppressor of T cell activation.

Nevertheless, the assertion that the Collisson–Exocrine and Bailey ADEX and Immunogenic subtypes were all associated with lower tumor purity cannot be entirely true, since the same gene expression signatures seen in patient clinical PDAC tumors are identified in derived cell lines—cell lines and xenografts from these same tumors, and specifically the Classical, Quasi-mesenchymal and Exocrine-like gene expression profiles (Jones et al., 2008; Knudsen et al., 2018). Moreover, most clinical PDAC tumors have low cellularity, so these too should be included to avoid observer bias. The Bailey Immunogenic subtype as well as containing gene expression profiles derived from tumor stroma immune infiltration predominantly related to B and T cells also contains an underlying Pancreatic progenitor-like gene expression character (Bailey et al., 2016). Both cytotoxic (CD8^+^) and regulatory T cells (CD4^+^CD25^+^FOXP3^+^ Tregs) are predominant (Bailey et al., 2016). It has been suggested that a distinct Immunogenic subtype does not exist as distinct since immune infiltrates are enriched across all tumor-intrinsic subtypes, and their prevalence is primarily driven by tumor cellularity of the sequenced samples (International Cancer Genome Consortium et al., 2010; Puleo et al., 2018; Maurer et al., 2019). Nevertheless, by allowing for different degrees of cellularity, the strong Pancreatic Progenitor-like signals can still be split into an immune high signature (Immunogenic subtype) and an immune low signature (Progenitor subtype) indicating that the signals from the underlying epithelium drive and formulate tumor cell immunogenicity (Figure 1; Bailey et al., 2016; Bear et al., 2020; Sahai et al., 2020). Hwang et al. using single-nucleus RNA sequencing found an association between Basal-like programs and higher immune infiltration with increased lymphocytic content, whereas Classical-like programs were associated with sparser macrophage-predominant microniches (Hwang et al., 2020).

At the present time, it is not entirely clear that the Collisson Exocrine-like and Quasi-Mesenchymal subtypes, and the Bailey ADEX and Immunogenic subtypes should be discarded, as there is a considerable variation in the way samples have been retrieved, stored, analyzed for mRNA expression, and assessed for epithelial cell purity by direct and indirect methodologies (Table 2; International Cancer Genome Consortium et al., 2010; Collisson et al., 2011; Carter et al., 2012; Moffitt et al., 2015; Bailey et al., 2016; Williamson et al., 2016; Cancer Genome Atlas Research Network, 2017; Connor et al., 2017; Raphael and Cancer Genome Atlas Research Network, 2017; Knudsen et al., 2018; Mateo et al., 2018; Puleo et al., 2018; Wartenberg et al., 2018; Maurer et al., 2019; Bear et al., 2020; Chan-Seng-Yue et al., 2020; Dijk et al., 2020; Escobar-Hoyos et al., 2020; Hong et al., 2020; Hwang et al., 2020). The validity of the Collisson Exocrine-like and Quasi-mesenchymal subtypes, and the Bailey ADEX and immunogenic subtypes requires further investigation.

### Commonality of Transcriptomic Signatures

**FIGURE 2:**
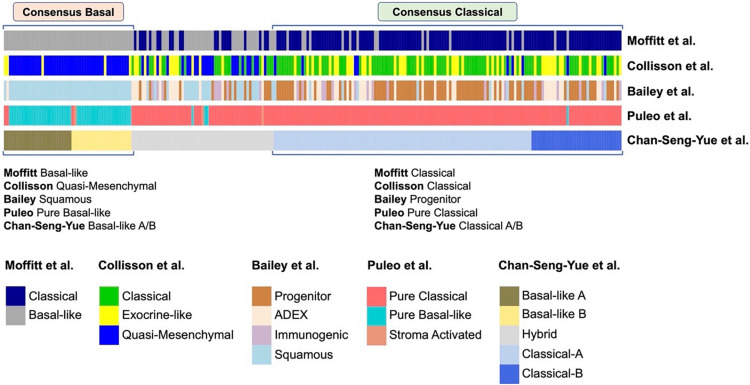
Comparison of different transcriptional classifications of PDAC. Comparison of previously published transcriptional classifications of PDAC, two major consensus subtypes have been identified (Chan-Seng-Yue et al., 2020): **(A)** Consensus Classical, which is named as “Classical” in the classifications of Collisson et al. (2011) and Moffitt et al. (2015), “Progenitor” by Bailey et al. (2016), “Pure Classical” by Puleo et al. (2018), and “Classical-A/-B” by Chan-Seng-Yue et al. (2020); **(B)** Consensus Basal, which is named as “Basal-like” by Moffitt et al. (2015), “Quasi-Mesenchymal” by Collisson et al. (2011), “Squamous” by Bailey et al. (2016), “Pure Basal-like” by Puleo et al. (2018), and “Basal-like A/B” by Chan-Seng-Yue et al. (2020).

There is a strong alignment between the Classical/Pancreatic Progenitor and Quasi-mesenchymal/Basal-like/Squamous subtypes signatures of Moffit, Collinson, Bailey, Puleo and Chan-Seng-Yue (Figure 2; Moffitt et al., 2015; Bailey et al., 2016; Cancer Genome Atlas Research Network, 2017; Puleo et al., 2018; Chan-Seng-Yue et al., 2020).

**Classical/Pancreatic progenitor** tumors have a better prognosis with pancreatic specific transcription factors, such as GATA6, PDX1, and HNF1A, that act to specify and maintain pancreatic identity.
**Basal-like/Squamous** tumors are associated with a poor prognosis, with increased mtKRAS allelic imbalance and changes in DNA methylation that ultimately repress pancreatic identity and activate characteristic multigene programs (International Cancer Genome Consortium et al., 2010; Lomberk et al., 2018; Puleo et al., 2018; Chan-Seng-Yue et al., 2020).

## Stromal Immune Cell and Cancer-Associated Fibroblast Infiltrate

Signals from the stroma play an important role in disease progression (Bailey et al., 2016; Ligorio et al., 2019; Bear et al., 2020; Sahai et al., 2020). PDAC is characterized by a complex and dense microenvironment with an extensive desmoplastic stromal reaction. Typically, around 5–30% of cells in pancreatic tumors are epithelial cancer cells. Activation of pancreatic stellate cells and cancer-associated fibroblasts (CAF), along with inflammatory and immune cell accumulation, occurs during early pancreatic tumorigenesis, creating an immunosuppressive microenvironment that restricts immune surveillance and supports tumor growth and invasiveness (Figure 1; Bear et al., 2020; Sahai et al., 2020). Oncogenic driver mutations promote immunosuppression from the earliest stages of tumor inception that accompanies oncogenesis. Beyond immunogenic prognostic subtypes, patient-specific immune changes should be considered in combination immune-modulatory therapies targeting roadblocks in antitumor immunity. An immune-signature-based stratification may guide personalized therapy of PDAC patients and enable the design of novel combinatorial treatments with improved clinical efficacy (Kandimalla et al., 2020).

Pancreatic cancer has relatively few coding mutations and thus only few neo-antigenic targets, and is embedded in an immunosuppressive cold tumor microenvironment, which impedes intratumoral CD8^+^ T cell infiltration and activation (Bear et al., 2020). Therefore, endogenous PDAC-reactive T cells are limited in quantity and quality and single agent immunotherapies with immune checkpoint inhibitors, which unleash pre-existing T cell immunity, are mostly ineffective in pancreatic cancer (Bear et al., 2020). Yet, exceptionally high neoantigen numbers, with robust antitumor CD8^+^ T cell responses have been associated with long-term survival in pancreatic cancer patients, and immune checkpoint blockade has shown clinical responses in patients with hypermutated MMR-deficient tumors (Balachandran et al., 2017; Le et al., 2017). To induce specific antitumor adaptive immune responses, tumor-derived antigens must not only be taken up by innate immune cells; they must also be efficiently processed and cross-presented to CD8^+^ T cells in the presence of a costimulatory signal. This mechanism is impeded in the vast majority of PDAC tumors by immunosuppressive mechanisms. CD8^+^ lymphocytes are trapped in peritumoral compartments and mostly display an exhausted gene expression profile (Steele et al., 2020). Th1-polarized CD4 + T cells are less frequent at the tumor site compared to Th2-polarized CD4^+^ T cells (De Monte et al., 2011). Tumor-associated macrophages (TAM) and myeloid-derived suppressor cells (MDSCs), which thwart the generation of cytotoxic T cell responses, are predominant over dendritic cells (DCs) that are largely dysfunctional, and immune checkpoint ligands are upregulated on myeloid cells (Hegde et al., 2020; Steele et al., 2020). In order to overcome the web of immune resistance and achieve durable antitumor effects, immunotherapeutic regimens need to target different steps in the cancer-immunity cycle, combining ideal antigen presenting cell (APC) activation that mediates priming of tumor-specific T cells, with strategies that enhance T cell effector function, and disrupt immunosuppressive myeloid cell programs. Thus, immune-modulatory strategies must be multi-modal aiming to (1) enhance endogenous T-cell function, (2) adoptively transfer tumor-specific T-cell immunity, and (3) attempt to devise an immunologically hot tumor microenvironment (Bear et al., 2020).

Moral et al. have shown that group 2 innate lymphoid cells (ILC2s) infiltrate PDACs to activate tissue-specific tumor immunity, inferring another novel immunoregulatory target (Moral et al., 2020). Enhanced anti-tumor immunity ensued blockade of the T cell checkpoint receptor programmed death (PD) receptor-1, which released ILC2 cell-intrinsic inhibition to expand and activate the tumor ILC2s to produce CCL5, thereby resulting in CD103^+^ dendritic cell expansion and then CD8 + T-cell activation (Moral et al., 2020). Tumor infiltrating ILC2s which express the programmed cell death protein (PD-1) receptor were enriched in long-term survivors with an immunologically hot tumor microenvironment containing abundant activated CD8^+^ T-cells, and containing higher bulk tumor RNA expression of the ILC2-activating cytokine IL33 (Moral et al., 2020). Pre-clinical studies in KPC mouse models have also suggested that specific targeting of macrophages and neutrophils using small molecule inhibitors, specific for either macrophage receptor CSF1R or the neutrophil receptor CXCR2, might facilitate better outcomes by enhancing endogenous T-cell cancer killing functions and the reprogramming of tumor cell intrinsic phenotypes (Steele et al., 2016; Candido et al., 2018). The PD-1 antibody pembrolizumab has FDA approval to for the treatment of MSI-H solid tumors, although this is present in only 1–3% of pancreatic cancers (Marcus et al., 2019; Marabelle et al., 2020). Inhibition of the CXCR4–CXCL12 pathway in pancreatic cancer also enhances tumor sensitivity to anti-PD-1 ligand-1 treatment. In the two-cohort phase IIa, COMBAT study (NCT02826486) pembrolizumab was combined with BL-8040 (a CXCR4 antagonist) in metastatic pancreatic cancer with promising responses and survival rates (Bockorny et al., 2020).

## Taking Molecular Subtyping Into Clinical Trials

Targeted therapies for advance pancreatic cancer based on next generation sequencing has been disappointing, with only 3–5% showing any clinical benefit in terms of actionable mutations, and limited to only a few months of additional survival (Pishvaian et al., 2020; Cobain et al., 2021). In reality the greatest sensitivity of pancreatic cancer to systemic therapies is chemotherapy, with increasing interest being shown in developing treatment response transcriptomic signatures to different agents (Tiriac et al., 2018). Moreover, it is important to distinguish treatment response signatures from the two main molecular subtypes as the Basal-like subtype appears to be more chemoresistant compared with reduced patient survival to the Classical subtype (Bailey et al., 2016; Aung et al., 2018; Chan-Seng-Yue et al., 2020; O’Kane et al., 2020). Several groups have now established informatic approaches that proport to accurately stratify patients based on PDAC subtype for clinical use. These include PuRIST, a single sample classifier that can stratify patients into two tumor-cell intrinsic subtypes based on the Moffitt classification scheme (Rashid et al., 2020). While PuRIST and other similar approaches promise better patient selection for chemotherapy, they have not been assessed in clinical trials.

Noll et al. identified hepatocyte nuclear factor (HNF)-1A and KRT81 that enabled stratification of tumors into different molecular subtypes by using immunohistochemistry (Noll et al., 2016). The two-marker combination identified the QM-PDA (KRT81^+^/HNF1A^–^) subtype, which was associated with the shortest survival; the Exocrine-like (KRT81^–^/HNF1A^+^) subtype which was associated with the longest survival; and the Classical (KRT81^–^/HNF1A^–^) subtype, which was associated with intermediate survival (Noll et al., 2016). Exocrine-like subtype tumors were resistant to tyrosine kinase inhibitors (erlotinib and dasatinib) and paclitaxel, which induced cytochrome P450 (CYP) 3A5 (CYP3A5) in the tumors, leading to the metabolism of these compounds (Noll et al., 2016). CYP3A5 expression was correlated positively with HNF1A^+^ and negatively with KRT81^–^, and also contributed to acquired resistance in the QM-PDA and Classical subtypes (Noll et al., 2016).

Hwang et al. (2020) performed single-cell RNA sequencing on 26 flash-frozen pancreatic cancers from patients who underwent surgical resection, with upfront surgery in 11 and in 15 after neoadjuvant chemoradiotherapy. Following chemoradiation, there was a relative increase in Basal-like cells (including the master transcription factor ΔTP63 for the Squamous subtype), and a decrease in Classical-like cells (including the hallmark transcription factor GATA6) (Hwang et al., 2020). Thus, there appears to be a commonality with the effects of chemotherapy such as FOLFIRINOX which will also enrich for the Basal-like subtype (Aung et al., 2018; Porter et al., 2019). Following chemoradiotherapy, there was enhanced expression of genes needed to maintain the Wnt/β-catenin niche, which is critical for treatment resistance and can be mediated by autocrine signaling of the epithelial cells and/or paracrine interactions with CAFs (Hwang et al., 2020). Squamous/Basal-like programs facilitate immune infiltration compared with the Classical-like programs (Hwang et al., 2020; Somerville et al., 2020). Importantly, the immune infiltrates associated with Basal-like and Classical-like malignant cells are distinct, pointing to differential strategies choosing checkpoint inhibitors for the Basal-like subtype, and for the Classical-like subtype choosing myeloid directed therapies such as CD40 agonists and TGF-β modulators (Hwang et al., 2020). The study by Hwang et al. (2020) has yet to be published following review and the findings and conclusions will need further evaluation. Other approaches to subtyping may be required to understand more fully the extent of interpatient heterogeneity such as differential DNA methylation, associated with interferon (IFN) signaling (Espinet et al., 2021).

The encouragement from COMBAT along with other immunotherapy approaches currently being tested will expand the armamentarium against pancreatic cancer. A detailed understanding of the individual patient’s response to the different forms of treatment will be necessary to further improve the prognosis of pancreatic cancer patients. The Bailey Immunogenic subtype is associated with immune gene programs involving B-cell signaling pathways, antigen presentation, CD4^+^ T-cell, CD8^+^ T-cell, and Toll-like receptor signaling pathways (Bailey et al., 2016). Acquired tumor immune suppression pathways through upregulation of the T cell checkpoint receptor PD-1 and cytotoxic T-lymphocyte-associated protein 4 (CTLA-4) in this Immunogenic subtype may offer therapeutic opportunities (Bailey et al., 2016). Puleo et al. (2018) found that the expression of CTLA-4 was higher in the Immune classical and Desmoplastic subtypes and, to a lesser extent, in the Pure basal-like subtypes, making these subtypes potentially sensitive to anti-CTLA-4 therapy such as ipilumumab. Also other promising therapeutic targets identified were the inhibitory checkpoint membrane receptors CD276 (B7-H3) and HAVCR2 (TIM3), both of which were highly expressed in the Desmoplastic, Stroma activated, and Pure basal-like subtypes (Wartenberg et al., 2018). Immune classical and Desmoplastic subtypes also showed high expression of the T-cell checkpoint inhibitor receptor CTLA-4, the costimulatory T-cell receptor CD27, and the tumor inhibitory T4^+^ cell subset CD26 marker protein; Basal-like tumors were enriched in CD276 (B7-H3) and HAVCR2 (TIM3); and PDL-2 (PDCD1LG2) was expressed in all but the Pure classical subtypes, which overall do not up-regulate any of the immune checkpoints (Puleo et al., 2018).

In an effort to improve outcomes in pancreatic cancer through the use of more effective therapeutics, large-scale efforts are required with multiple centers and cooperating disciplines. Multiple clinical trials have been launched, pursuing better treatment schemes and ideal medication regimens based on molecular profiling and subtyping including COMPASS, PREDICT-PACA, Precision Promise, Know Your Tumor, ESPAC6/7, PANCuRx, and PASS1 (Table 3).

**TABLE 3:** Translational clinical trials with molecular subtyping.

**Trial name**	**Year**	**Type of study**	**Country**	**Tumor stage**	**Methodology**	**Treatment**	**Aims/Results**
COMPASS (Aung et al., 2018; O’Kane et al., 2020) NCT02750657	2018	Cohort study	Canada	Locally advanced or metastatic PDAC	RNA-seq	156 Classical vs. 39 Basal-like subtypes, 91 mFFX vs. 63 GnP	ORR: 33% vs. 10%; Classical mFFX 29.6%, Basal-like mFFX 10%, Classical GA 39%, Basal-like GA 11%. mOS: 9.3 vs. 5.9 mths; Classical mFFX 10.6 mths, Basal-like mFFX 6.5 mths, Classical GA 8.19 mths, Basal-like GA 8.12 mths.
ESPAC-6 EudraCT: 2020-004906-79	2021	Phase III	Europe	Resectable PDAC	LCM, organoid generation, RNA-seq, WGS/WES	Adjuvant chemotherapy, mFFX vs. Gemcitabine with capecitabine	Patients randomized to allocation of oxaliplatin- or gemcitabine-based chemotherapy by standard clinical criteria or by a transcriptomic treatment specific stratification signature after surgery. (1) Primary outcome: disease free survival, time from randomization to disease recurrence or death from any cause (2) Secondary outcomes: overall survival; survival based on targeted signatures in test versus control arms; response to targeted therapies
ESPAC-7	2022	Randomized phase II	Europe	Non-metastatic locally advanced PDAC	Organoid generation, RNA-seq, WGS/WES	Neoadjuvant chemotherapy, mFFX vs. GnP	Patients randomized to allocation of oxaliplatin- or gemcitabine-based chemotherapy by standard clinical criteria or by a transcriptomic treatment specific stratification signature before surgery. (1) Primary outcome: resection rate following neoadjuvant therapy Disease free survival, time from randomization to disease recurrence or death from any cause (2) Secondary outcomes: resection rates and disease-free survival based on targeted signatures in test versus control arms; overall survival; response to targeted therapies
PASS-01 NCT04469556	2019	Randomized phase II	United States, Canada	Metastatic PDAC	Organoid generation, RNA-seq	mFFX vs. GA	(1) To determine the PFS benefit, ORR, DOR, and OS of mFFX compared to GA in patients with metastatic PDAC. (2) To evaluate chemotherapy sensitive signatures. (3) To determine if organoid transcriptomic profiles parallel patient transcriptomic profiles. (4) To explore biomarkers as responders to mFFX/GA (including GATA6 validation).
Know Your Tumor (Pishvaian et al., 2020)	2020	Registry study	USA	Pancreatic cancer	Genomic testing and tailored treatment recommendation based on the molecular profiling.	Molecularly matched therapy group vs. unmatched therapy group vs. no molecular marker group	mOS 2.58 vs. 1.51 vs. 1.32 y; patients with 2/more lines of therapy: mOS 1.81 vs. 0.85 vs. 0.73 y, mPFS 10.93 vs. 4.53 vs. 5.37 mths;
Precision Promise NCT04229004	2020	Phase II/III trials	United States	Metastatic PDAC	Genomic, transcriptomic and immune analysis	SM-88 vs. SOC (GA/mFFX)	(1) To compare each investigational arm in OS in 1st/2nd line metastatic pancreatic cancer patients and to determine which patients benefit from which regimen. (2) To determine short-/long-term safety signals of each investigational arm. (3) To determine clinical benefit (PFS, OS, CR, and PR) and the duration of it.
PREDICT-PACA DRKS00022429	2021	Co-clinical validation cohort study	Germany	Metastatic PDAC	Organoid generation/chemosensitivity testing, PDX analyses, NGS, transcriptomics,	Gemcitabine monotherapy, GnP, 5-FU with oxaliplatin	(1) To validate molecular signatures predicting response to specific chemotherapy regimens and establish point-of-care diagnostics. (2) To establish PDO-based chemosensitivity testing for individual tumors. (3) To evaluate the influence of the tumor microenvironment’s composition on chemotherapy response.
PANCuRx	2021	Cohort studies	Canada	PDAC	WGS, RNA-seq	Translational Research Initiative	(1) To seek solutions to the high fatality rate of PDAC by generating genetic and biologic knowledge of the cancer, the mechanisms of how tumors grow and tailored treatment options. (2) To expand the COMPASS trial into the other cancer centers across Canada.

*COMPASS, Study of Changes and Characteristics of Genes in Patients with Pancreatic Cancer for Better Treatment Selection; ESPAC, European Study Group for Pancreatic Cancer; PASS-01, Pancreatic Adenocarcinoma Signature Stratification for Treatment; PREDICT-PACA, Integrated Biopsy-Based Approach to Predict Response to Chemotherapy for Patients with Stage IV Pancreatic Cancer; NGS, next-generation sequencing; mFFX, modified FOLFIRINOX; GnP, gemcitabine plus nab-paclitaxel; GA, gemcitabine-abraxane; SOC, standard of care; mths, months; y, year; ORR, overall response rate; (m)OS, (median) overall survival; PFS, progression-free survival; DOR, duration of response; CR, complete response; PR, partial response; PDO, patient-derived organoid.*

The EPPIC (Enhanced Pancreatic Cancer Profiling For Individualized Care Study) study based in Canada aims to sequence metastatic pancreatic tumors of 400 patients through two clinical trials (COMPASS and PanGen), both of which are generating molecular and phenotypic signatures of individual tumors in a clinically relevant timeframe and related to chemotherapy responses^1^ [Aung et al., 2018; O’Kane et al., 2020].

Precision Promise was created by the Pancreatic Cancer Action Network and 15 USA clinical academic sites, in cooperation with the FDA and pharmaceutical partners. Precision Promise is an active adaptive Phase II/III clinical trial platform (NCT04229004) that allows rapid evaluation of novel therapeutic options in patients with metastatic pancreatic cancer. The protocol utilizes an adaptive randomization design and includes several trial designs and statistical innovations. All patients undergo pre- and on-treatment biopsies with state-of-the-art genomic, transcriptomic, and immune analysis, along with collection of blood research samples throughout the study. Focused on both first- and second-line treatment of metastatic PDAC, 30% of patients are randomized to one of the two common control arms (gemcitabine plus nab-paclitaxel and FOLFIRINOX), while 70% of patients are randomized to an experimental treatment arm. The platform currently has one experimental arm open (SM-88, Tyme), with two additional experimental arms to be added in 2021. Compared to traditional trial designs, Precision Promise has several advantages: multiple investigational treatments can be evaluated simultaneously using common controls; only 175 patients per experimental arm are required to initiate a regulatory registration, and it is expected that this platform will significantly accelerate the time to evaluate a new therapy, with an anticipated cost savings of 30–50%. With its unique design and novel method of data sharing, Precision Promise serves as a new clinical trial ecosystem to accelerate drug development for PDAC.

The ESPAC-6 adjuvant trial in resectable patients and the ESPAC-7 neoadjuvant trial in locally advanced patients are evaluating oxaliplatin- or gemcitabine-based chemotherapy response of PDAC patients that will be randomized according to standard clinical criteria (control arms) or by transcriptomic stratification signatures (experimental arms).

The PREDICT-PACA co-clinical trial funded by the German Cancer Aid is being conducted as a biopsy-based approach to predict response to chemotherapy for patients with metastasized pancreatic cancer. The consortium has established robust and highly predictive transcriptomic signatures linked to specific chemotherapy response profiles. Using microfluidic card-based qRT-PCR marker panels, clinical utility of the signatures is validated in a prospective collection of core biopsies from metastases of stage IV PDAC patients that receive one of the current chemotherapies upon clinical decision. In parallel, potential alternative therapies for third-line treatment are identified by high-throughput drug screening using patient-derived organoids and by next-generation sequencing-based detection of actionable mutations.

A major area of discussion is whether to discard the tumor microenvironment for molecular classification and replace this with a tumor-cell intrinsic classification system, but this may seem short-sighted as the stroma is a uniquely powerful biological phenomenon in pancreatic cancer. The relationship between molecular subtypes, most notably the immunogenic subtype as described by Bailey et al. (2016), and the responsiveness to evolving multimodality and immunotherapeutic strategies needs further investigation. Single-cell and spatial transcriptomic approaches now allow single-cell profiling of tumor and immune cell populations resident in patient tumors (Kuboki et al., 2019; Hwang et al., 2020; Moncada et al., 2020). These approaches are providing unparalleled insights into immune-tumor interactions and offer new opportunities for targeted immunotherapeutic intervention.

## Author Contributions

XZ, KH, PB, CS, SR, TG, MB, and JN prepared the initial drafts of the manuscript which were then critically reviewed by RK, BB, JA, KW, TP, MWB, and TH. All of the authors approved the final version of the manuscript.

## Conflict of Interest

XZ is in receipt of funding from the Chinese Scholarship Council; PB declares grants from Horizon Europe 2020 (Marie Skłodowska Curie Innovative Training Network); SR declares grants from German Cancer Aid grants (70112720 and 70113167); JN declares grants from the Dietmar Hopp Stiftung GmbH, the Stiftung Deutsche Krebshilfe, and the Heidelberger Stiftung Chirurgie. The remaining authors declare that the research was conducted in the absence of any commercial or financial relationships that could be construed as a potential conflict of interest.

## Publisher’s Note

All claims expressed in this article are solely those of the authors and do not necessarily represent those of their affiliated organizations, or those of the publisher, the editors and the reviewers. Any product that may be evaluated in this article, or claim that may be made by its manufacturer, is not guaranteed or endorsed by the publisher.

## Footnotes

1 https://www.tfri.ca/our-research/research-project/enhanced-pancreatic-cancer-profiling-for-individualized-care-(eppic)
