# Corrigendum: Clinical impact of molecular subtyping of pancreatic cancer

**DOI:** 10.3389/fcell.2023.1179559

**Published:** 2023-03-16

**Authors:** Xu Zhou, Kai Hu, Peter Bailey, Christoph Springfeld, Susanne Roth, Roma Kurilov, Benedikt Brors, Thomas Gress, Malte Buchholz, Jingyu An, Kongyuan Wei, Teresa Peccerella, Markus W. Büchler, Thilo Hackert, John P. Neoptolemos

**Affiliations:** ^1^ Department of General, Visceral and Transplantation Surgery, Heidelberg University Hospital, Heidelberg, Germany; ^2^ Section of Surgical Research, Heidelberg University Hospital, Heidelberg, Germany; ^3^ Institute of Cancer Sciences, University of Glasgow, Glasgow, United Kingdom; ^4^ Department of Medical Oncology, National Center for Tumor Diseases, Heidelberg University Hospital, Heidelberg, Germany; ^5^ Division of Applied Bioinformatics, German Cancer Research Center, Heidelberg, Germany; ^6^ Department of Gastroenterology and Endocrinology, Philipps University of Marburg, Marburg, Germany

**Keywords:** molecular subtypes, transcriptomes, structural variants, precision medicine, next generation sequencing, clinical trials, ESPAC

In the published article, an author name was incorrectly written as Zhou Xu. The correct spelling is “Xu Zhou”.

The authors apologize for this error and state that this does not change the scientific conclusions of the article in any way. The original article has been updated.

